# Percutaneous Catheter Drainage in Acute Infected Necrotizing Pancreatitis: A Real-World Experience at a Tertiary Care Hospital in North India

**DOI:** 10.7759/cureus.27994

**Published:** 2022-08-14

**Authors:** Satwant Singh, Siddharth Prakash, Deepak Kaushal, Honey Chahal, Ajit Sood

**Affiliations:** 1 Interventional Radiology, Dayanand Medical College and Hospital, Ludhiana, IND; 2 Surgery, Sant Sarwan Dass Charitable Hospital Kathar, Kathar, IND; 3 Gastroenterology, Dayanand Medical College and Hospital, Ludhiana, IND

**Keywords:** acute necrotizing pancreatitis, real world scenario, percutaneous catheter drainage, open necrosectomy, infection

## Abstract

Introduction

Open necrosectomy in acute infected necrotizing pancreatitis is associated with very high mortality and morbidity. Moreover, if it is performed before four weeks, the benefits are limited. In this study, we evaluated the safety and efficacy of percutaneous catheter drainage (PCD) in patients with acute infected necrotizing pancreatitis.

Methods

It was a single-center, observational study, where all consecutive patients with proven or probable infected acute necrotizing pancreatitis in whom PCD was performed were studied. The patients who failed to respond to PCD underwent open necrosectomy. Baseline characteristics and the outcome of all included patients, including complications of PCD, were studied.

Results

A total of 46 patients (males=36, females=10) underwent PCD over a period of 18 months. Fifteen (32.60%) patients succumbed to their illness. PCD benefitted a total of 31 (67.39%) patients; in 17 (36.95%) patients, it worked as a standalone therapy, while in 14 (30.43%) patients, additional surgery was required where it helped to delay the surgery. Median days at which PCD and surgery were performed were 17.5 days (range: 2-28 days) and 33 days (range: 7-70 days), respectively. Lower mean arterial pressure at presentation, presence of multiorgan failure, more than 50% necrosis, higher baseline creatinine and bilirubin levels, and an early surgery were markers of increased mortality. Three (6.5%) patients had PCD-related complications, out of which only one required active intervention.

Conclusion

PCD in infected acute pancreatic necrosis is safe and effective. In one-third of the patients, it worked as standalone therapy, and in the rest it delayed the surgery beyond four weeks, thereby preventing the complications associated with early aggressive debridement.

## Introduction

Acute necrotizing pancreatitis constitutes 10-20% of all acute pancreatitis and has high morbidity and mortality [1,2]. Pancreatic and peri-pancreatic collections may remain sterile or can become infected; however, there is no co-relation between the extent of necrosis and risk of infection [3-6]. Diagnosis of infection in pancreatic collections is extremely important as infected pancreatic collection warrants addition of antibiotics and a possible active intervention [3]. The infection may be diagnosed if gas is seen in pancreatic and peri-pancreatic tissues on contrast-enhanced computed tomography (CECT) or if the imaging-guided fine needle aspiration of collection (FNAC) reveals bacteria or fungi on gram stain or culture [7]. The PANTER study has already established the superiority of “step-up approach” in the management of acute infected necrotizing pancreatitis. New-onset multiple organ failure, incisional hernias, and secondary diabetes were lower in the group assigned to step-up approach [8], which consisted of an initial percutaneous drainage (PCD) or endoscopic drainage of necrotic tissue. Patients were subjected to minimal invasive retroperitoneal necrosectomy only if PCD failed. In this study, we evaluated the safety and efficacy of PCD in infected acute pancreatic collection in real-world scenario.

## Materials and methods

This study was an observational study conducted over a period of 18 months (January 2014 to June 2015) at a tertiary care hospital in North India. All consecutive patients who had acute necrotizing pancreatitis with proven or probable infection and who underwent PCD were included. Ethical clearance to conduct the study was taken from the Institutional Ethical Committee vide DMCH/R&D/2014/56 dated 18/1/14. The infection was diagnosed on the basis of CECT findings (presence of gas in the fluid collection) or if there was evidence of bacterial/fungal infection on gram stain or culture in the imaging-guided FNAC of collection [7]. Patients who had clinical deterioration, high leucocyte count with rising procalcitonin, and lactate levels despite the optimal intensive care, in the absence of other focus of infection, were considered to have probable infected acute necrotizing pancreatitis. Modified CT severity index (CTSI) score was calculated using pancreatic inflammation, pancreatic necrosis, and extra-pancreatic complications on CECT and ranged from 0-10, with 10 being the most severe pancreatitis. Systemic inflammatory response syndrome (SIRS) was defined as two or more out of the following four: temperature >38.0°C or <36.0°C, heart rate > 90 beats/minute, respiratory rate >20 breaths/minute, leukocytosis > 12,000/dL, or leucopenia < 4,000/dL. The patients with a duration of acute pancreatitis beyond four weeks, acute exacerbation of chronic pancreatitis, pancreatic pseudocyst, walled-off necrosis, and pancreatic malignancy were excluded. Clinical profile of included patients, response to PCD, and any complications arising due to the placement of drainage catheter were noted.

Procedure technique

All PCD procedures were performed under either computed tomography (CT) or ultrasound (US) guidance. Access routes that avoided the colon, small bowel, stomach, liver, spleen, and kidney were selected to minimize the risk of bacterial contamination, hemorrhage, and internal organ injury. As the collections in acute necrotizing pancreatitis are often viscous, catheters with multiple side holes with a minimum diameter of 12-14 French (F) were introduced into the collections using the Seldinger technique. Multiple catheters (maximum: 3) were placed in the fluid collections depending on their location and extent.

Catheter monitoring

Catheters were irrigated with 20 mL of normal saline at least thrice a day. Monitoring of catheter output was done on a daily basis. If the catheter was not draining or the patient was having persistent sepsis, US or CT was performed to reassess the residual collections, and flushing or upsizing of the catheter was performed as required. The catheter was removed if there was no residual collection on follow-up CT/USG, and output was less than 10 mL/day for two consecutive days. PCD was considered effective if there was control of sepsis (defervescence of fever and return of inflammatory markers to normal) and resolution of necrotic collections. If there was no clinical improvement after 72 hours of drain placement, an imaging (US/CECT) was performed to check the position of the catheter and the same was re-adjusted if indicated. In the absence of any additional drainable collections and after ruling out any other source of infection, the patient was taken up for surgery (open necrosectomy), in case there was a deterioration of at least two organ systems (circulatory, pulmonary, or renal), or at least 10% deterioration of two out of three parameters: leucocytes/temperature/C-reactive protein [8].

Statistical analysis

Appropriate statistical tests were used for the quantitative and qualitative data. Quantitative data were described as mean ± standard deviation and median, depending on the distribution. Pearson chi-square test and Fisher’s exact tests were applied to discrete variables, as applicable. A p-value (two-sided) of less than 0.05 was considered significant.

## Results

A total of 46 patients (males=36, females=10) with proven or probable infected acute necrotizing pancreatitis who underwent PCD were studied during the study period (January 2014 to June 2015). The mean age of the cohort was 43.22 ± 15.06 years. The etiology of acute pancreatitis was excessive alcohol intake in 21 (45.6%) patients, gallstone disease in 13 (28.2%) patients, drug-induced (valproic acid) in 1 (2.2%) patients, while 11 (23.9%) patients were considered to have idiopathic acute pancreatitis. A pre-procedure FNAC was performed in six patients. The most common organisms isolated in our patients were gram-negative bacilli (61%) (*Klebsiella pneumonia* [31.4%], *Escherichia coli* [21.6%], *Acinetobacter boumannii* [15.7%], *Enterobacter cloacae* [7.8%], and *Proteus mirabilis* [3.9%]). In rest of the patients, infection was polybacterial (39%). Table 1 describes the baseline characteristics of our cohort.

**Table 1:** Baseline characteristics of the cohort CTSI, computed tomography severity index; PaO_2_, partial pressure of oxygen); PCD, percutaneous catheter drainage; SIRS, systemic inflammatory response syndrome

Characteristics	n = 46
Median age, years (range)	42 (18-80)
Male sex (%)	36 (78.26)
Etiology of pancreatitis	
Gall stone disease, n (%)	13 (28.26)
Alcohol, n (%)	21 (45.66)
Drug induced, n (%)	1 (2.17)
Idiopathic, n (%)	11 (23.91)
Modified CTSI	
Median	10
Range	4-10
Extent of necrosis	
<30%, n (%)	19 (41.30)
30-50%, n (%)	15 (32.60)
≥50%, n (%)	12 (26.10)
Central necrosis (%)	19 (41.30)
Disease severity	
SIRS, n (%)	40 (86.95)
Single-organ failure, n (%)	18 (39.13)
Multiple-organ failure, n (%)	7 (15.21)
Mean white cell count × 10−9/liter ± SD	16.88 ± 6.57
Mean creatinine (mg/dL) ± SD	1.21 ± 0.98
Mean calcium (mg/dL) ± SD	7.82 ± 1.06
Mean random blood sugar (mg/dL) ± SD	164.6 ± 82.96
Mean PaO_2_ (mm Hg) ± SD	65.52 ± 15.05
Mean arterial pressure (mm of Hg) ± SD	112 ± 14.75
Median days of pancreatitis at which PCD was performed	17.5 (range: 2-28)
Median days of pancreatitis at which surgery was performed	33 (range: 7-70)
Median days of hospital stay	25.5 (range: 3-88)

**Figure 1:**
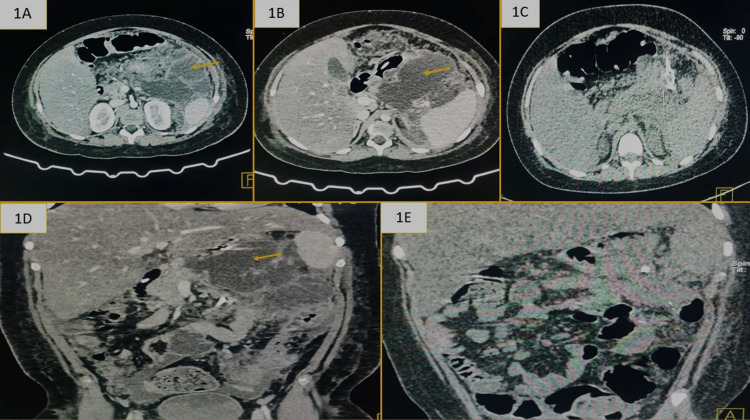
Computed tomography image of one of our patients (A, B) Preprocedural axial CECT images showing well-defined collection involving the distal body and tail of the pancreas extending till anterior pararenal space and large collection in the lesser sac compressing the greater curvature of the stomach. (C) Post-percutaneous catheter drainage axial CT image showing significant reduction in the peripancreatic collection. (D) Pre-procedural coronal CECT showing large collection in the peripancreatic region and left anterior pararenal space. (E) Post-procedural coronal CT shows drainage tube in situ with significant reduction in collection. CT, computed tomography

**Figure 2:**
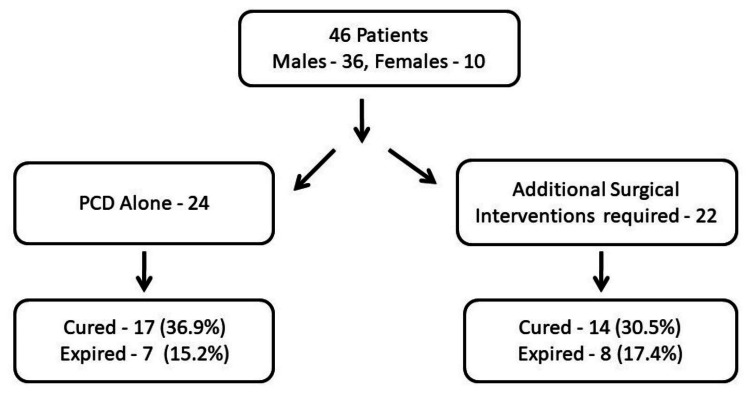
The cohort PCD, percutaneous catheter drainage

Out of a total of 46 patients, 31 (67.39%) patients survived. While 17 (36.95%) of them required only PCD, 14 (30.43%) patients required a subsequent surgery. A total of 15 (32.60%) (36.95%) succumbed to their illness. Figure 1 represents the CT image of one of our patients who underwent successful PCD. Figure 2 illustrates our cohort, the interventions done, and the outcome. The patients who had resolution of pancreatitis and discharged home were referred to as cured. Three (6.52%) patients had PCD-related complications, one (2.17%) patient had bowel perforation, and two (4.34%) patient had intra-peritoneal hemorrhage. The patient with perforation required left hemicolectomy, and those with hemorrhage were managed conservatively as bleeding ceased spontaneously without any hemodynamic compromise. No blood transfusion was required in either of the two patients. There was one incidence of accidental pullout of catheter by the patient without any clinical consequence.

The median days at which PCD and surgery were performed were 17 days (range: 2-28) and 33 days (range: 18-70) of onset of pancreatitis, respectively. The median of hospital stay was 25.5 days (range: 3-88 days). The patients who had a fatal outcome were more likely to be females (p-value: 0.005) and had lower mean blood pressure at presentation (101.46 ± 15.22 vs. 117.03 ± 11.69 mm of Hg, p-value: 0.0004) and multiorgan dysfunction (p-value: 0.0013). Extent of necrosis was a predictor of patient outcome; among patients with extent of necrosis, less than 30% had a mortality rate of 21.05% (4/19 patients), those with necrosis of 30-50% had a mortality rate of 20 % (3/15 patients) and those with >50 % necrosis had a mortality rate of 75% (9/12 patients) (p-value: 0.043). The presence of central necrosis was a significant factor affecting cure rates; 9/19 (47.36%) patients with central necrosis were cured, while 11/13 (84.61%) patients with non-central necrosis were cured (p-value: 0.049).

Amylase levels were higher in patients who succumbed to their illness (827.53 ± 927.51 units/liter vs. 371.93 ± 413.82 units/liter; p-value: 0.025); however, the lipase levels were not significantly different in two groups. Post-PCD, higher total leucocyte count (14.69 ± 6.18 x 10^3^ per dL vs. 11.58 ± 3.51 x 10^3^ per dL, p-value: 0.034), higher creatinine (1.71 ± 1.23 vs. 0.60 ± 0.32, p-value: <0.0001), and higher bilirubin levels (1.89 ± 2.53 vs. 0.76 ± 0.44, p-value: 0.018) were present in patients who died due to pancreatitis. The median days of pancreatitis at which surgery was conducted were significantly higher in patients who survived (34.5 days [range: 25-70 days] vs. 27 days [range: 18-49 days], p-value: 0.04). Tables 2, 3 summarize and compare the findings in patients who survived and succumbed to their illness.

**Table 2:** Univariate analysis of the patients who survived and those who succumbed to the illness CTSI, computed tomography severity index; GSD, gall stone disease; PaO_2_, partial pressure of oxygen; PCD, percutaneous catheter drainage;  TLC, total leucocyte count

	Alive (n=31)	Expired (n=15)	p-Value
Median age in years	44 (range: 18-80)	41 (range: 22-70)	0.632
Sex (male/female)	29/2	9/6	0.005
Etiology			
Alcohol, n (%)	16	5	0.248
GSD, n (%)	8	5	0.598
Drug induced, n (%)	0	1	0.152
Idiopathic, n (%)	7	4	0.763
Mean PaO_2_, mm Hg (± SD)	67.06 ± 15.29	62.33 ± 14.50	0.322
Mean arterial pressure, mm Hg	117.03 ± 11.69	101.46 ± 15.22	0.0004
Median CTSI score	9 (Range: 6-10)	8 (range: 4-10)	0.984
More than 50% necrosis (n)	3	7	0.0047
Mean TLC (x 10^3 ^per dL) (± SD)	Pre-PCD: 18.24 ± 7.20	Pre-PCD: 14.06 ± 3.89	0.052
	Post-PCD: 11.58 ± 3.51	Post-PCD: 14.69 ± 6.18	0.034
Single organ dysfunction, n	11	7	0.471
Multiple organ dysfunction, n	1	6	0.0013
Mean creatinine (mg/dL) (± SD)	Pre-PCD: 1.07 ± 0.79	Pre-PCD: 1.50 ± 1.28	0.167
	Post-PCD: 0.60 ± 0.32	Post-PCD: 1.71 ± 1.23	<0.0001
Mean bilirubin (mg/dL) (± SD)	Pre-PCD: 1.65 ± 1.78	Pre-PCD: 2.06 ± 1.51	< 0.0001
	Post-PCD: 0.76 ± 0.44	Post-PCD: 1.89 ± 2.53	0.018
Mean amylase (units/liter)(± SD)	371.93 ± 413.82	827.53 ± 927.51	0.025
Mean lipase (units/liter) (± SD)	599.61 ± 980.69	1149.8 ± 1574.19	0.152
Mean calcium (mg/dL) (±SD)	Pre-PCD: 7.95 ± 0.99	Pre-PCD: 7.57 ± 1.19	0.259
	Post-PCD: 8.15 ± 0.33	Post-PCD: 8.16 ± 0.63	0.943
Mixed flora, n	10	6	0.608
Mean number of drains (±SD)	1.74 ± 0.73	1.66 ± 0.49	0.703
Repositioning, n	7	4	0.763
PCD as standalone therapy/PCD followed by surgery	17/14	7/8	0.60
Median days of pancreatitis at which PCD was inserted	19.5 (range: 6-28 days)	16 (range: 2-18 days)	0.152
Median days of pancreatitis at which surgery was conducted	34.5 (range: 25-70 days)	27 (range: 18-49 days)	0.04
Median hospital stay	28 (range: 10-50 days)	23 (range: 3-43 days)	0.101

**Table 3:** Multivariate analysis of variables pf the patients who survived and those who succumbed to their illness CI, confidence Interval; CTSI, computed tomography severity index; GSD, gall stone disease; PaO_2_, partial pressure of oxygen; PCD, percutaneous catheter drainage;  TLC, total leucocyte count

	Alive (n=31)	Expired (n=15)	p-Value
Sex (male/female)	27/4	9/6	0.057
Mean arterial pressure, mm Hg (± SD)	117.03 ± 11.69	101.46 ± 15.22	0.001
	95% CI: 112.74-121.32	95% CI: 93.04-109.9	
	Range: 80.0-130.0	Range: 80.0-120.0	
Organ failure			0.073
Multiorgan failure	1 (8.33%)	6 (46.15%)	
Single organ failure	11 (91.67%)	7 (53.85%)	
Serum amylase	371.94 (± 413.82)	827.53 (± 927.51)	0.174
	95% CI: 220.15-523.73	95% CI: 313.89-1341.17	
	Range: 21.0-2176.0	Range: 20.0-3374.0	
TLC after PCD	11.58 (± 3.51)	14.69 (± 6.18)	0.111
	95% CI: 10.3-12.87	95% CI: 11.27-18.12	
	Range: 7.6-20.9	Range: 6.1-24.6	
Creatinine after PCD	0.605 (± 0.317)	1.71 (± 1.23)	<0.001
	95% CI: 0.489-0.722	95% CI: 1.03-2.39	
	Range: 0.27-1.9	Range: 0.32-5.1	
Total bilirubin after PCD	0.761 (± 0.442)	1.89 (± 2.54)	0.071
	95% CI: 0.598-0.923	95% CI: 0.483-3.29	
	Range: 0.26-2.18	Range: 0.27-9.57	
Day of pancreatitis on which surgery was performed	39.14 (± 11.78)	29.38 (± 9.35)	0.026
	95% CI: 32.34­-45.94	95% CI: 21.56-37.19	
	Range: 25.0-70.0	Range: 18.0-49.0	

The patients who survived, but required additional surgery were more likely to have higher bilirubin post-PCD (1.89 ± 2.53 vs. 0.65 ± 0.33 mg/dL, p-value: 0.05) and to have polymicrobial flora (p-value: 0.0081). Median days at which PCD was performed in whom additional surgery was required was significantly more than the patients in whom only PCD was sufficient for the cure (median: 23 days [range: 10-28 days] vs. 14 days [range: 6-18 days], p-value: 0.035). Table 4 compares the patients who required only PCD vs. ones who required subsequent surgery. However, on multivariate analysis, none of the variables significantly predicted additional surgery.

**Table 4:** Survivors who required only PCD vs. those who required subsequent surgery CTSI, computed tomography severity index; PCD, percutaneous catheter drainage; TLC, total leucocyte count

	Only PCD and alive (n=17)	PCD and surgery and alive (n=14)	p-Value
Median CTSI score	8 (Range: 6-10)	10 (Range: 6-10)	0.726
More than 50% necrosis	1	2	0.438
TLC (x10^3 ^per dL)	Pre-PCD: 17.45 ± 5.51	Pre-PCD: 19.18 ± 8.97	0.514
	Post-PCD: 11.44 ± 3.18	Post-PCD: 11.57 ± 3.98	0.920
Single organ dysfunction	5	6	0.443
Multiple organ dysfunction	1	0	0.364
Creatinine (mg/dL)	Pre-PCD: 1.14 ± 0.98	Pre-PCD: 0.97 ± 0.48	0.558
	Post-PCD: 0.61 ± 0.36	Post-PCD: 0.60 ± 0.26	0.931
Total bilirubin (mg/dL)	Pre-PCD: 1.59 ± 1.88	Pre-PCD: 2.06 ± 1.51	0.456
	Post-PCD: 0.65 ± 0.33	Post-PCD: 1.89 ± 2.53	0.05
Amylase	277.47 ± 190.04	486.64 ± 569.99	0.165
Lipase	452.70 ± 738.93	778 ± 1218.17	0.366
Calcium (mg/dL)	Pre-PCD: 7.75 ± 1.08	Pre-PCD: 8.18 ± 0.85	0.235
	Post-PCD: 8.15 ± 0.33	Post-PCD: 8.19 ± 0.37	0.752
Mixed flora	2	8	0.0081
Number of drains (mean)	1.82 ± 0.63	1.64 ± 0.84	0.500
Repositioning	5	2	0.323
Median days of pancreatitis at which PCD was inserted	14 (range: 6-18)	23 (range: 10-28)	0.035
Hospital stay	19 (range: 10-44)	39 (range: 25-88)	<0.0001
Median days of pancreatitis at which surgery was conducted	-	35.5 (range: 25-70)	-

**Figure 3:**
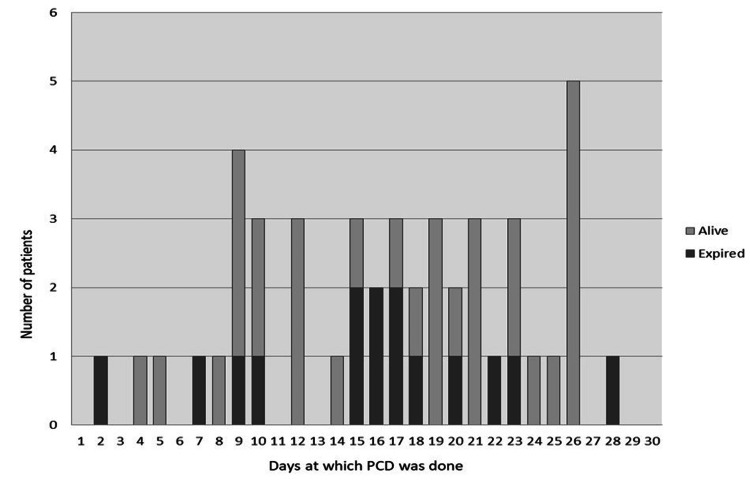
Number of days at which PCD was performed and the outcome PCD, percutaneous catheter drainage

As for clinical success, the 18th-day threshold remained the most significant factor; out of 26 patients who required drainage in ≤18 days, 11 expired with an overall mortality rate of 42.30%. On the other hand, only four (20%) out of 20 patients who required drainage after day 18 succumbed to their illness. However, in the rest 15/26 (57.69%) patients, only three (11.53%) patients required additional surgery and the rest 12 (80%) patients were cured by PCD alone. Figure 3 depicts the outcome and day of PCD. Of the six patients who underwent surgery during the first 28 days, five (83.3%) patients expired and only one (16.7%) patient had a complete recovery, whereas among the 16 patients who underwent surgery after 28 days, 13 (81.2%) patients recovered and only three (18.8%) patients expired. The findings were statistically significant with a p-value of 0.014.

## Discussion

Severe acute pancreatitis carries a high mortality with two distinct peaks. During the initial first week, the mortality occurs due to persistent multiorgan dysfunction as a result of SIRS [9-11]. Beyond the first week, local complications such as necrosis and infection further add to the disease severity. Mortality associated with multiorgan failure in acute pancreatitis during the initial days may be as high as 36%-50% [9,12,13], which can be further increased if a patient develops infected necrosis [8,14]. Over the years, open necrosectomy has been considered the primary treatment for infected pancreatic necrosis [15-17], but it is associated with significant mortality and morbidity [18]. This led to the use of step-up approach, which involves percutaneous or endoscopic drainage of necrotic tissue followed by minimally invasive retroperitoneal necrosectomy, if required. The purpose of drainage is to control the source of sepsis, which may delay or obviate the need for subsequent necrosectomy. Van Santvoort et al. in their landmark paper showed that the step-up approach in infected pancreatic necrosis results in not only lesser incidence of new onset multiorgan dysfunction but also lower incidence of diabetes mellitus on longer follow-up [8]. Bakker et al. compared surgical versus endoscopic drainage of pancreatic collections and found that complication rates including new onset organ failure is much lesser with endoscopic approach [19]. Akshintala et al. compared percutaneous versus endoscopic approach and found the latter superior to the percutaneous approach in terms of re-intervention rates, number of follow-up imaging, and total hospital stay [20]. A recent multicentric randomized controlled trial compared endoscopic approach to surgical step-up approach and found the former no superior to the latter in terms of mortality and major complications. However, the total hospital stay and rate of pancreatic fistula were lower with endoscopic approach [21].

We had an overall mortality of 32.60%, which was higher than reported in other series (around 17%) [22]; however, as in the PANTER trial [8], 36.9% of our patients could be successfully managed with PCD alone and did not require any further intervention. The mean hospital stay of the patients cured with PCD alone was 19 days (range: 10-44 days), while the minimal hospital stay as reported in the literature after upfront surgery in infected necrosis is around one month [8]. Twenty two of our patients required additional surgery, out of which 17 survived. In patients whom PCD was not effective as a standalone therapy, PCD helped in delaying the surgery and acted as a bridge to definitive therapy. The median days at which the surgery was performed was significantly higher in patients who survived (34.5 days (range: 25-70 days) vs. 27 days (range: 18-49 days), p-value: 0.004, reemphasizing the fact that necrosectomy should be delayed as long as possible to allow the demarcation between the necrotic and healthy tissue [23]. Rodriguez et al. in their study including patients with acute necrotizing pancreatitis found that operative mortality was much higher in patients who were operated on before 28 days (20.3% vs. 5.1%, p = 0.002) [24]. Mier et al. [25] reported a mortality of 56% (3.4 times the control) in the group who underwent early debridement, leading to early termination of the trial. Similar to the study conducted by Baudin et al. [26], in the index study, the patients who required PCD before 18 days of onset of acute pancreatitis had lower chances of survival. Nonetheless, only 3/15 patients who survived with early PCD required additional surgery. Hence, when dealing with an early infected pancreatic necrosis (≤4 weeks), a step-up approach with PCD is a much better alternative. Not only it helps in delaying the surgery but may also be a standalone therapy.

More than 50% necrosis predicted mortality in our patients, and a similar trend has been noted by Freeny et al. [27] and Pal et al. [28]. As documented in our study, central necrosis is associated with less than optimal outcomes with PCD. In the study conducted by Freeny et al., only four out of 14 patients with central necrosis benefitted from PCD [27]. Central necrosis is usually associated with disruption of the middle portion of the main pancreatic duct, which effectively isolates the head of the pancreas from the body and tail. It leads to fistulous communication between the duct and collection, which requires a distal pancreatectomy and cannot be managed with PCD alone. The other factors that predict failure of PCD as standalone therapy are higher post-PCD bilirubin levels and the polymicrobial infection. Only three patients had PCD-related complications, out of which one patient required active intervention.

Limitations

The limitation of the study is that the sample size is relatively small, and it is an observational study with no control group. Since it is a single-center study, the expertise of intervention radiologist and surgeon may have altered the results. The patients were not followed up for long-term complications such as secondary diabetes mellitus and pancreatic insufficiency.

## Conclusions

In this study, we evaluated the role of PCD in the management of acute infected pancreatic necrosis in a real-world scenario and found the approach to be both safe and effective. Besides delaying the surgery in critically ill patients, PCD may act as a standalone therapy in one-third of the patients. The drainage of infected necrosis may be the only option in patients who present with infected acute pancreatic necrosis early in course of disease.
